# An Approach to Improve Energy Efficiency during Antimicrobial Blue Light Inactivation: Application of Pulse-Width Modulation Dimming to Balance Irradiance and Irradiation Time

**DOI:** 10.3390/antibiotics12091431

**Published:** 2023-09-11

**Authors:** Wanqing Zhang, Ping Su, Jianshe Ma, Ying Tan, Mali Gong, Liya Ma

**Affiliations:** 1 Tsinghua Shenzhen International Graduate School, Tsinghua University, Shenzhen 518055, China; zhangwq21@mails.tsinghua.edu.cn (W.Z.); su.ping@sz.tsinghua.edu.cn (P.S.); gongml@mail.tsinghua.edu.cn (M.G.); 2 Department of Precision Instrument, Tsinghua University, Beijing 100084, China; 3 Shenzhen Baoan Women and Children’s Hospital, Jinan University, Shenzhen 518100, China; maria226@sina.com

**Keywords:** antimicrobial blue light, energy efficiency, pulse blue light, disinfection, pulse-width modulation

## Abstract

Antimicrobial blue light (aBL) is an effective non-destructive inactivation technique and has received increasing attention. Despite its significance, the existing research has not thoroughly delved into the impacts of irradiance and irradiation time on enhancing energy efficiency during aBL inactivation and the explanation of the enhancement effect of pulse exposure. In this paper, a series of *Escherichia coli* inactivation experiments with different duty cycles, pulse frequencies, and irradiation times were conducted, and the relative concentrations of reactive oxygen species (ROS) were measured under corresponding conditions. A two-dimensional (2-D) Hom model was proposed to evaluate the effect of irradiance and irradiation time. The results show that, compared to continuous exposure, pulsed aBL (duty cycle = 25%) can save ~37% of the energy to achieve the same inactivation effect and generate a 1.95 times higher ROS concentration. The 2-D Hom model obtains the optimal combination of average irradiance and time according to the desired reduction and shows that the irradiation time has a higher weight than the irradiance (1.677 and 1.083, respectively). Therefore, using pulse exposure with a lower average irradiance for a longer period of time can achieve a better inactivation effect when consuming equivalent energy. The proposed pulse-width modulation dimming approach helps promote the application of the aBL technique.

## 1. Introduction

Light-based disinfection techniques have the advantage of a wide range of pathogen inactivation techniques without reducing antibiotic resistance [1,2]. Ultraviolet (UV) light leads to the formation of pyrimidine dimers and consequently cellular damage, which is the most widely used light-based disinfection technique [3]. However, UV is not suitable for the disinfection of space where human activities are present due to its carcinogenic effect and limited penetration distance [4,5]. Recently, the light-emitting diode (LED) with 400 nm–470 nm antimicrobial blue light (aBL) has been considered as an effective non-destructive inactivation technique and received increasing attention [6]. The principle of aBL inactivation is generally accepted such that some endogenous photosensitizers (PS) (e.g., porphyrins and flavins [7]) can absorb photon energy in the aBL wavelength band and generate reactive oxygen species (ROS), which can subsequently inactivate bacteria [8,9]. Among various ROS, singlet-state oxygen is regarded as the main toxic substance [10,11]. The complex photochemical reactions involved in singlet-state oxygen generation are described in our previous studies [12].

A considerable number of studies have investigated aBL inactivation effects on different microbes [13,14,15] and built models of inactivation kinetics to fit the light dose and bacterial reduction, including the modified Chick–Watson model [16], Weibull model [17], and Hom model [16]. In practical applications, aBL is a good prospect because of its longer travel distance than UV light and its non-destructive nature to cells. For example, it can be used as a ceiling-mounted light system in clinical environments to reduce bacterial contamination of environmental surfaces [18,19] and as an approach to burn wound treatment [20,21], gonorrhea treatment [22], and plasma disinfection [23]. It also shows potential in food sterilization (such as milk [24], meat [25], and fruit [26,27,28]), which can remove microorganisms while preserving flavor. Some methods have been proposed to improve aBL’s energy efficiency which refers to the capacity to inactivate microorganisms per unit of energy. Based on the mechanism, these methods can be divided into two categories. One is considering the synergistic inactivation of aBL with other environmental conditions, such as temperature [29], humidity [30], and pH [31]. The other is about the irradiation approach, such as exploring aBL that better matches the absorption peak wavelength of PS [32].

However, there is a lack of exploration on how to balance irradiance and the irradiation time to improve the energy efficiency in the existing aBL studies. Numerous researchers have proved that the inactivation effect improves as the irradiation dose increases. As is well-known, the irradiation dose is the combination of irradiance and time. Both higher irradiance and longer time can enhance inactivation. Nevertheless, it is unclear whether higher irradiance or longer time should be applied for energy conservation purposes. Most studies have limitations on experimental equipment, where irradiance is fixed and the irradiation dose is only varied by varying the time. 

The irradiance of LEDs can be changed by adjusting the drive current or the duty cycle of the pulse-width modulation (PWM). The former method will result in a limited dimming range, decreased luminous efficiency, and color shift in LEDs [33], while PWM dimming has been widely used for driving LEDs due to its fast response and linear dimming [34,35]. Adjusting the duty cycle of PWM, the peak irradiance in every single cycle remains constant, whereas the output average irradiance is regulated in accordance with varying duty cycles. While changing the pulse frequency has no effect on the average irradiance and peak irradiance, employing PWM can provide a simple way to vary the irradiance.

In aBL inactivation, only a very small number of studies have explicitly used PWM to generate a pulse light for inactivation. Gillespie et al. [36] initially demonstrated that pulsed 405 nm blue light improved the efficiency compared to continuous exposure in a fixed irradiation time. The inactivation effect of longer wavelengths (≥450 nm) of pulse blue light was also studied [30,37,38]. Nevertheless, these studies mainly focus on the improvement effect of pulse blue light compared to continuous exposure and lack the analysis of energy efficiency. Moreover, the study on the mechanism to explain the enhancement effect of pulse exposure is absent from the current research. 

The contributions of this work are threefold. Firstly, a PWM signal-controlled experimental setup with adjustable uniform irradiance and constant temperatures was established. Secondly, the inactivation experiments on *Escherichia coli* were conducted under different duty cycles and pulse frequencies. The viability of the bacteria was measured. The ROS generation for different duty cycle–time combinations at the same irradiation dose was probed. Finally, the energy efficiency was defined and calculated under different experimental conditions. A two-dimensional (2-D) Hom model was proposed to fit the irradiance-time-reduction data, evaluate the effect of irradiance and irradiation time, and calculate the optimal set of conditions according to the desired reduction. In this paper, it is concluded that a low average irradiance for a long time can obtain a higher energy efficiency during inactivation, which is conducive to the practical application of aBL technology and energy conservation.

## 2. Results

### 2.1. Measurement of Irradiance and Temperature at Different Duty Cycles

**Figure 1:**
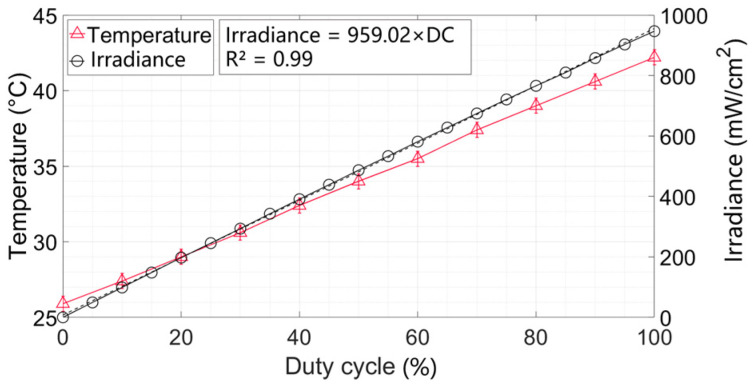
Irradiance and temperature at different duty cycles.

Figure 1 shows the measured irradiance and temperature within 1 h of the LED treatment at different duty cycles. The irradiance and temperature sensors were placed 5 cm below the LED light source, which is the same distance as the inactivated sample from the light source. When the duty cycle was 100% (i.e., continuous exposure), the measured irradiance was 946.64 mW/cm^2^ (i.e., the peak irradiance). The duty cycle can be changed to produce the desired average irradiance according to Equation (6) and the fitted peak irradiance was 959.02 mW/cm^2^ (R^2^ = 0.99). Under the peak irradiance, the temperature rose to 42.3 ± 0.1 °C (16.3 °C above room temperature), at which temperature *Escherichia coli* can still survive although the growth dynamics slow down [39].

### 2.2. Investigating the Effects of the Duty Cycle on Energy Efficiency

To investigate the inactivation under different duty cycles of aBL treatment, five duty cycles (12.5%, 25%, 50%, 75%, and 100%) were studied. The reduction in *E. coli* was evaluated at different irradiation times. In all the experiments, the pulse frequency was fixed at 100 Hz.

**Figure 2:**
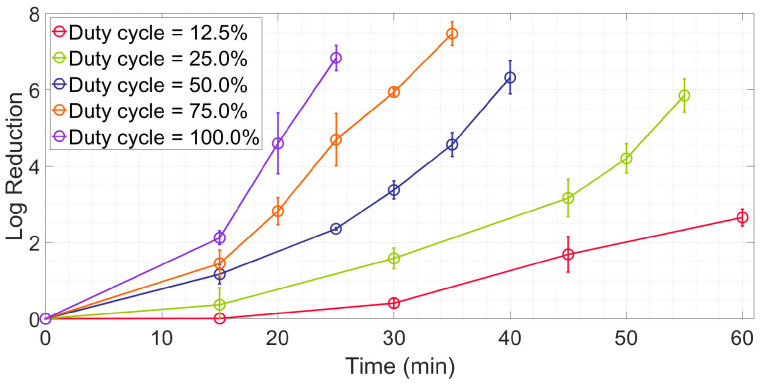
Log reduction vs. irradiation time for different duty cycles.

As shown in Figure 2, the inactivation pattern is similar for different duty cycles, which demonstrates a non-linear relationship. The pattern is consistent with previous studies in *E. coli* inactivation [31,40]. The reduction in *E. coli* grew with an increasing irradiation time and duty cycle because the irradiation dose received by the *E. coli* increased. The inactivation speed (the slope of every curve) was slow at the beginning and then accelerated in all five curves. When the irradiation time = 15 min, the reduction in the $DC_{100\%}$ was 2.12 log, which was 1.82 times larger than that in the $DC_{50\%}$. For the irradiation time = 25 min, the corresponding values were 6.83 log and 2.90 times. With the increase in irradiation time, the difference in the reduction increased more prominently.

**Figure 3:**
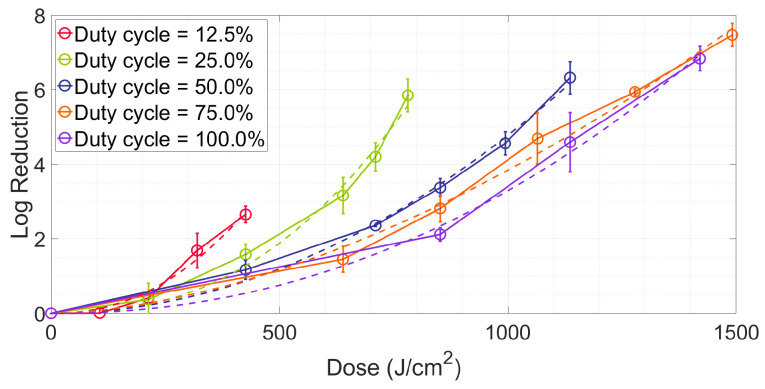
Log reduction vs. irradiation dose for different duty cycles.

*E. coli* is more sensitive to aBL with a low duty cycle and less irradiation is required to achieve the same reduction. Figure 3 shows the relationship between the reduction and irradiation dose. The photodynamic inactivation curves [41] fitted by the Hom model (Equation (10)) at different duty cycles are also demonstrated in Figure 3. For the same irradiation dose, a lower duty cycle light achieves a higher reduction. According to the fitting inactivation kinetic parameters (as shown in Table 1), the irradiation doses needed to achieve 3 and 5 log reductions (D_3d_ and D_5d_) were calculated [13]. The aBL with a duty cycle = 12.5% resulted in ~54% dose conservation (according to Equation (9)) compared to continuous exposure. For the aBL with a duty cycle = 25%, the corresponding values were 37% and 39%.

**Figure 4:**
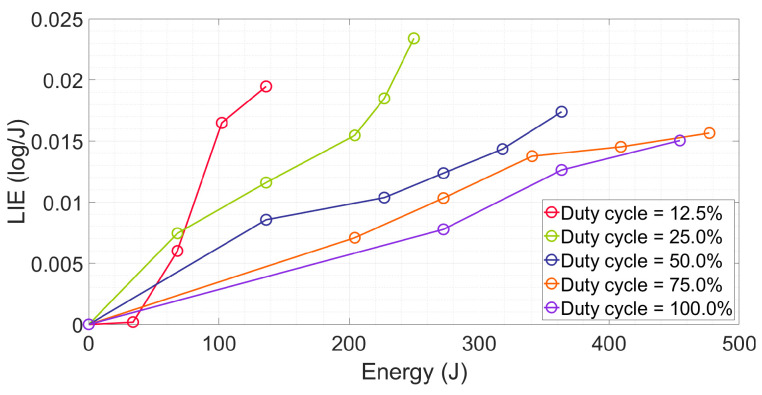
*LIE* vs. energy for different duty cycles.

The pattern of the *LIE* (defined as the log *reduction* per unit of energy (log/J) in Equation (8)) is not only related to the duty cycle but also to energy. As shown in Figure 4, for the same duty cycle, the *LIE* increased with the energy, corresponding to the acceleration of inactivation. The smaller the duty cycle, the higher the energy efficiency. When the energy = 136.3 J (the highest value for the duty cycle = 12.5%), the *LIE* of the $DC_{12.5\%}$ was 0.019 log/J, which was 1.68, 2.27, 3.89, and 4.86 times compared to the $DC_{25\%}$, $DC_{50\%}$, $DC_{75\%}$, and $DC_{100\%}$, respectively. It is worth noting that when the *LIE* of the $DC_{12.5\%}$, at an irradiation time of less than 15 min (i.e., energy less than 34.0 J), the *E. coli* was hardly inactivated.

### 2.3. Investigating the Effects of Pulse Frequency on Inactivation

Pulse frequency is another crucial parameter in PWM dimming. According to previous studies, the effect of pulse frequency on inactivation can be diverse under different duty cycles [36]. Thus, the *E. coli* reduction at different pulse frequencies was evaluated under $DC_{25\%}$, $DC_{50\%}$, and $DC_{75\%}\;$(the average irradiance was 174, 345, and 511 mW/cm^2^, respectively).

**Figure 5:**
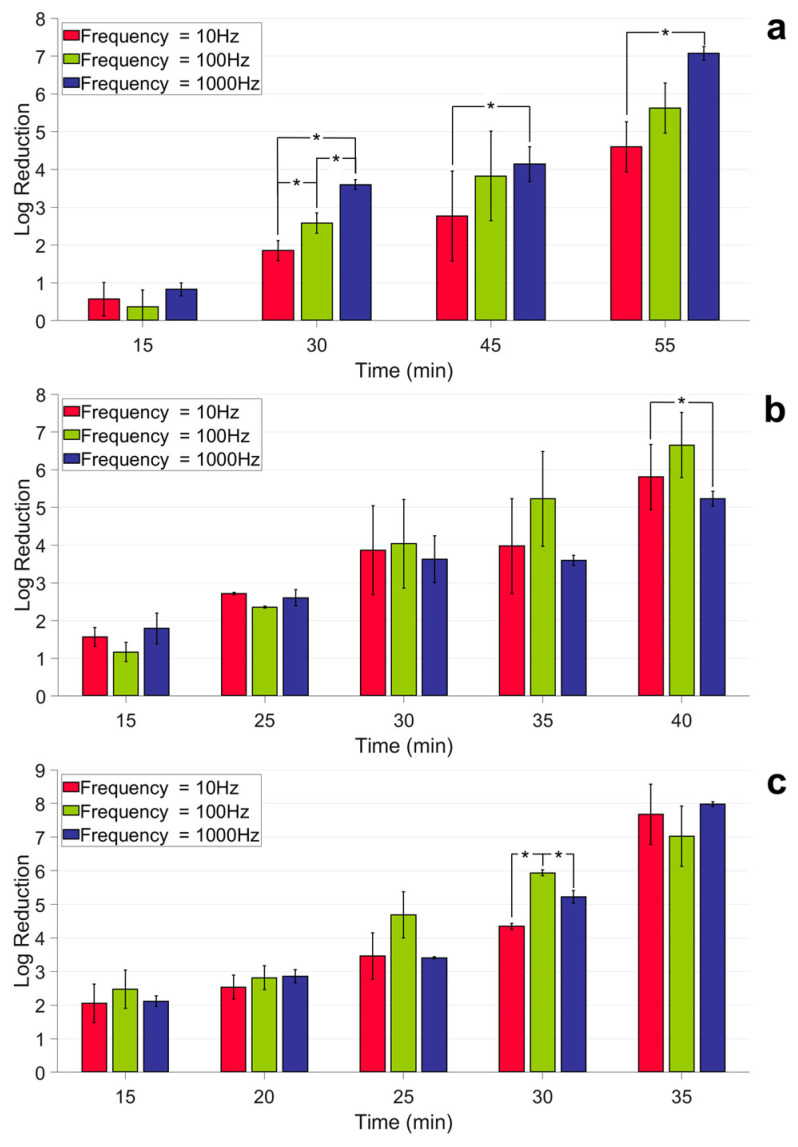
Log reduction vs. irradiation time for different pulse frequencies. (**a**) Duty cycle = 25%; (**b**) duty cycle = 50%; (**c**) duty cycle = 75%. Asterisks indicate significant differences between the groups (*, *p* < 0.05).

For the $PF_{10Hz}$ and $PF_{1000Hz}$ individually (as shown in Figure 5a–c), the reduction in bacteria increased with an increasing irradiation time as well as duty cycle, as in the previous analysis of the $PF_{100Hz}$ case in Section 2.2. However, there were differences in the impact of frequency on the inactivation under different duty cycles. For the $DC_{25\%}$ (as shown in Figure 5a), a significant difference (*p* < 0.05) was observed between the groups after 30 min of irradiation. The pulse frequency will enhance the reduction. Meanwhile, for the experiments with a $DC_{50\%}$ and $DC_{75\%}$ (as shown in Figure 5b,c), the variability was not significant between the different pulse frequencies. The pulse frequency may not affect inactivation under these duty cycles.

### 2.4. Fitting Irradiance-Time-Reduction Data Based on the 2-D Hom Model

**Figure 6:**
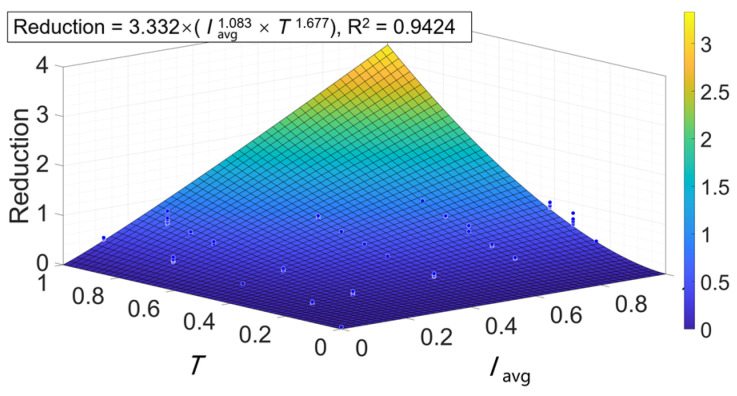
Three-dimensional perspective view of the effect of irradiance and irradiation time on reduction.

The 2-D Hom model in Equation (12) was proposed to fit the reduction data obtained above. The input data were normalized to remove the unit of the original data. As shown in Figure 6, the 2-D Hom model provided a good fitting surface with R^2^ = 0.9424. The exponent of the average irradiance I was 1.083 and the exponent of the irradiation time T was 1.677. It indicates that the effect of T on the inactivation has a higher weight. Changing the same step, T will cause a larger change in the reduction. Extending the irradiation time can improve energy efficiency.

Calculating according to Equation (14) and reversely normalizing, it can be obtained that if the desired reduction $R_{d}$ = 3, the optimal combination for irradiance and irradiation time is 90 mW/cm^2^ and 71.43 min, and the corresponding irradiation dose is 385.72 J/cm^2^. The value is lower than all the D_3d_ results in Table 1. This result is also supported by data from previous studies. Leanse et al. [42] used aBL with I = 100 mW/cm^2^ to inactivate *E. coli*. Fitting the reduction data by the Hom model can calculate that D_3d_ = 425.61 J/cm^2^, which is higher than our model. Dos Anjos et al. [43] applied 38.4 mW/cm^2^ and 180 min which only achieved a 2.84 log reduction, where the irradiation dose was 412.56 J/cm^2^. The validity of our model is preliminarily demonstrated, i.e., a suitable combination of irradiance and irradiation time can be found to achieve the desired reduction. It is worth noting that for different microorganisms and inactivation environments, there can be various weights of I and T, but it is possible to find an optimal combination by Equation (14).

### 2.5. ROS Generation at the Same Energy

**Figure 7:**
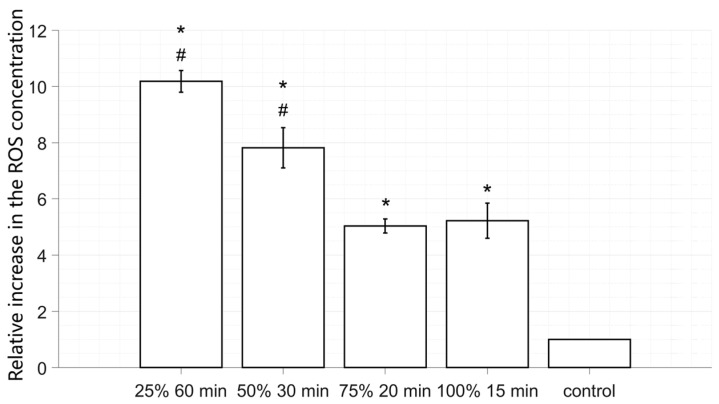
The measured relative ROS concentration data. Asterisks indicate significant differences vs. the control group (*, *p* < 0.05). # indicates significant differences vs. the continuous group (#, *p* < 0.05).

Lower irradiance combined with a longer irradiation time can generate more ROS. Four different duty cycle–time combinations were selected for the evaluation of ROS generation, as shown in Figure 7. The aBL irradiation dose in all four groups was 851.98 J/cm^2^. The group with a $DC_{25\%}$ and $T_{60min}$ generated the highest ROS concentration, which was 1.95 times higher than the group with a $DC_{100\%}$ and $T_{15min}$ (*p* < 0.01). Meanwhile, for the group with a $DC_{50\%}$ and $T_{30min}$, the value was 1.50. The difference in the ROS concentration between the group with a $DC_{75\%}$ and $T_{20min}$ and a $DC_{100\%}$ and $T_{15min}$ was not significant (*p* > 0.05). The ROS were all significantly increased in the four groups compared to the non-irradiated control group (*p* < 0.01).

## 3. Discussion

For light-based disinfection techniques, improving energy efficiency is conducive to its widespread application. Existing aBL studies have improved inactivation efficiency by applying optimal wavelengths and external conditions. There is a need to develop more effective irradiation methods for aBL by balancing the irradiance and irradiation time, as performed in the UV inactivation field [44,45]. In this study, a series of inactivation experiments were carried out to explore the sensitivity of *E. coli* to different duty cycles and pulse frequencies under different irradiation times. A 2-D Hom model was proposed for the first time to fit the obtained irradiance-time-reduction data. The method of using PWM signals to generate the desired irradiance can be easily integrated into existing light source systems, providing a simple approach to enhance inactivation, which will help to promote the aforementioned practical application of aBL and other circumstances.

**Figure 8:**
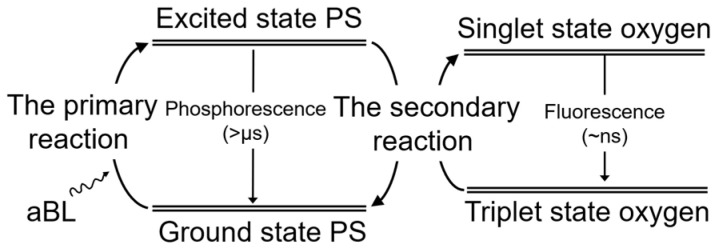
Photochemical reactions involved in aBL inactivation.

At an equivalent peak irradiance, employing pulse exposure can yield a more effective inactivation outcome under the same dose, as shown in Figure 3. We deduce that the reason may be attributed to the process of ROS generation. As in previous studies, a ROS is generated by multiple photoreactions [46]. As shown in Figure 8, the primary photochemical reaction is the stimulation of PS with photons and the secondary reaction is the conversion of triplet-state oxygen to singlet-state oxygen and other ROS by excited PS molecules, with this process being independent of light exposure [47]. Noteworthily, the occurrence of the photochemistry reactions (femtoseconds level) is much shorter than the lifespan of excited PS molecules which is at a microsecond level [48]. It may infer that the mismatch of the reaction rate will require some extra time for the excited PS molecules to reset to their ground state. Upon implementing continuous exposure, the primary and the secondary reactions both continuously proceed. However, it is possible that the primary reaction happens only partially, because some PS molecules are at their excited state. By comparison, with pulsed exposure, the primary and the secondary reactions are stimulated in the turned-on time of a pulse cycle (minimum at 250 microseconds). While in the turned-off time, the excited PS molecules are probably allowed to sufficiently reset in preparation for subsequent activation; meanwhile, the partly secondary reaction can continue to progress. This may avoid some unnecessary energy waste because both the reset process and the secondary reaction do not require light.

It may be possible to optimize phototoxicity by adjusting the pulse width. ROS generation primarily relies on three factors: the light condition, the type of photosensitizer, and the concentration of oxygen [22]. Other pertinent factors include the oxygenation level [47] and the rate of photobleaching of the chromophore that catalyzes the ROS formation [49]. In this work, we fixed the microbe species (the photosensitizers and chromophores included in the same species of *E. coli* are considered to be the same) and the experimental environments (a similar oxygen level). The variable in this study is the pulse width of the exposure light, which leads to different inactivation results. Among the five duty cycles selected in this paper, the lower the duty cycles (i.e., the shorter the turned-on time), the more significant the improvement in energy efficiency, as shown in Figure 4. In Section 2.5, the generation of ROS at the same irradiation dose was probed, which confirmed our above inference. More ROS generation suggests a higher inactivation rate can be achieved [22]. In other words, it is probable to achieve higher energy efficiency by allocating the $t_{on}$ time to an appropriate shorter period, that is, adjusting the pulse width as well as the average irradiance.

Another work is that a 2-D Hom model was proposed, which considered the effects of both irradiance and time on inactivation in the aBL field for the first time. According to Equation (6), the average irradiance is affected by the peak irradiance and duty cycle. Gillespie et al. [36] changed the duty cycle along with the peak irradiance to ensure the average irradiance remained stable, and the results showed that there was no significant difference in the reduction. This indicates that it is the average irradiance that affects the inactivation. Given this, the proposed model will be greatly helpful in practical applications. Through our model, the optimal combination of the average irradiance and time can be obtained according to the desired reduction, as shown in Section 2.4. Although the peak irradiance of existing light sources varies, the required average irradiance can be easily attained through the PWM signal. 

However, there is a minimum irradiance and dose threshold for the occurrence of lethal effects. For the experiment with the duty cycle of 12.5%, aBL did not achieve significant inactivation within 15 min (as shown in Figure 2). This phenomenon is consistent with the results of Dong et al. [50]. The lethal effect of ROS is reflected through its cumulative concentration (as shown in Equation (1)). Irradiance is the slope factor of the singlet--state oxygen accumulation rate. The death of bacteria needs to meet two thresholds. The irradiance threshold means that the ROS accumulation rate needs to exceed the self-scavenging rate [51,52], and the dose threshold means that the accumulation time needs to exceed the ROS concentration threshold that causes cell damage. Short exposure times within a single cycle may result in the deficiency of the dose and the produced singlet-state oxygen will be removed by bacterial cells. Therefore, a too-low average irradiance may not result in effective inactivation, even if the irradiation time is long enough. The conclusions and the calculation result in Section 2.4 hold only after the irradiance threshold is exceeded.

(1) dO2 1rxdt=fκ×Φ×[S0]×S∆×O 32/γ+S∆κ/ξ+ωη×[S0][β+O 32/γ+1]×(S∆κ/ξ+ωη×[S0])

where O2 1rx is the concentration of the cumulative singlet-state oxygen, in units of μM; Φ is the irradiance, in units of W/cm^2^. The other symbols appearing in the above equation were defined in the literature [12].

A general conclusion about the effect of the pulse frequency on inactivation is not available. Theoretically, ROS generation by aBL is an electron transfer reaction (type I) or energy transfer reaction (type II) [47]. As previously analyzed, the pulse frequency does not change the irradiance and dose and consequently has no effect on inactivation. The experimental group with the duty cycle of $DC_{50\%}$ and $DC_{75\%}$ verifies the theory. However, for the $DC_{25\%}$ group, using a higher frequency will obtain a better inactivation effect. It can be inferred that the width of the pulses may produce some impact on the organelles. The width of a single pulse was 250 microseconds at $DC_{25\%}$ and $PF_{1000Hz}$, which may result in some photophysical effects of the aBL [53], such as powerful photon strikes [13] and impulsive stimulated Raman scattering [54]. On the other hand, the influence of frequency on the inactivation effect might be related to the wavelength, irradiance, the species of inactivated microbe, etc., which would produce a synergistic effect. Gillespie et al. [36] found that 1 kHz had the best *Staphylococcus aureus* inactivation effect. Li et al. [55] regarded that 100 Hz was the optimal inactivation frequency in the study of UVA-LED. However, for UVC-LED, there is no clear relationship between frequency and efficiency [56]. Overall, it can be concluded that it is better to use a higher frequency (at least greater than the human eye’s retention frequency of 100 Hz) in the specific application of aBL (e.g., a combination with existing indoor lighting sources [18]), considering the comfort of the human eye.

In this paper, although the experimental results show that long-term irradiation with low irradiance can obtain higher energy efficiency, more experiments are needed to be conducted to verify the calculated optimal combination of the irradiance and irradiation time to achieve various desired reductions. The synergistic effect between the frequency and duty cycle should also be studied further. In practical applications, the cost of an extra pulsed system should be taken into account. While considering the maturity of PWM dimming technology for LEDs and the long-term cost of electricity, we believe that the use of a pulsed system is desirable. In addition, lower irradiance and pulse exposure will increase the time cost, because the turned-off time is required to be considered. In the future, LEDs with higher power can be used to increase the peak irradiance of a single cycle, so that the accumulation rate of ROS is accelerated, and both the time cost and energy conservation can be considered.

## 4. Materials and Methods

### 4.1. Bacterial Strains Preparation

The *E. coli* MG1655 (ATCC. 700926) used in this study was purchased from the American Type Culture Collection (ATCC; Manassas, VA, US) and stored at −80 °C. The bacteria were subcultured on Lysogeny broth agar (LBA; HuanKai Microbial, Guangzhou, China) plates at 37 °C for 24 h. A single colony was then inoculated into 30 mL of sterile Lysogeny broth (LB; HuanKai Microbial) and incubated with shaking at 180 rpm for 12 h at 37 °C. This working culture was then centrifuged at 2500 rpm for 5 min and the cell pellet was washed twice with phosphate-buffered saline (PBS; pH 7.4; HuanKai Microbial). The pellet was then resuspended in PBS to produce a concentrated cell culture (approximately 10^9^ colony-forming units (CFU)/mL).

### 4.2. Experimental Setup

#### 4.2.1. LED Treatment Setup

**Figure 9:**
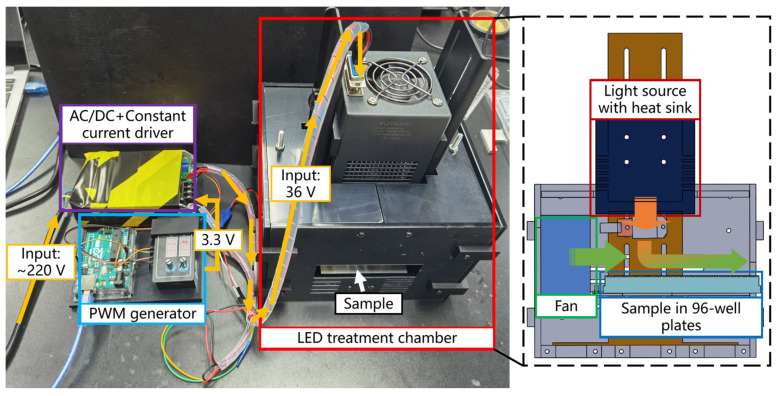
The aBL LED treatment setup.

The whole LED treatment setup (as shown in Figure 9) included the LED treatment chamber and the circuit section. An LED light source with a heat sink (FUTANSI, Shanghai, China) was mounted on top of the chamber. The LED chips were arranged in a 6 × 6 array in a 1 cm × 1 cm square. The distance between the LED and the sample was 5 cm. A fan was mounted on the side of the chamber to ensure that the temperature inside the chamber was stable during the irradiation process. The chamber was made of black acrylic glass and black resin to block unnecessary external light and reflection. The drive and control system was composed of a constant current driver and a PWM generator. The PWM generator provided driving signals to control the duty cycle (0–100%) and pulse frequency (1 Hz–1000 Hz) of the current output from the constant current source.

#### 4.2.2. Optical Parameters of the Light Source

**Figure 10:**
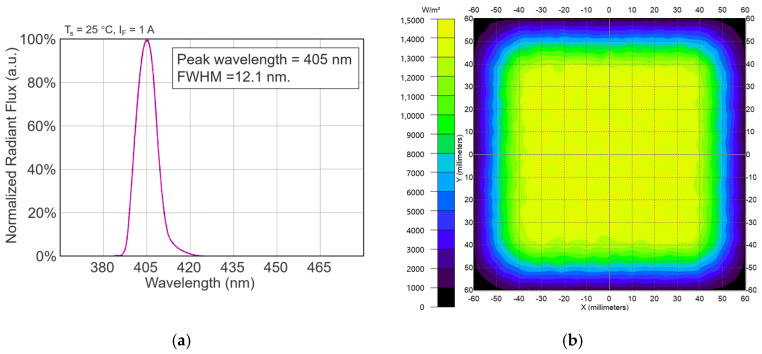
Optical parameters of the LED light source. (**a**) Normalized radiant flux of LED chip; (**b**) simulated irradiance distribution.

The LED chips used in the experiment had a peak wavelength of 405 nm and an FWHM (full width at half maximum) of 12.1 nm. The absolute spectral, as measured by a HAAS-2000 spectrometer (EVERFINE, Hangzhou, China), was normalized with respect to its integral over the range of the measured wavelength and depicted in Figure 10a. The light source in this experiment was able to provide uniform irradiance over a square area of 8 cm × 8 cm. Figure 10b shows the simulated irradiance distribution with a relative variation of less than 10%, which was calculated by Zemax OpticStudio v18.9 (ZEMAX Development Corporation, Kirkland, WA, USA). The actual irradiance was measured and confirmed by a PM100D power meter with an S121C probe (Thorlabs, Newton, NJ, USA), as shown in Section 2.1.

### 4.3. Definition of Variables

#### 4.3.1. Inactivation Quantification

The inactivation effect of *E. coli* was analyzed by calculating the log *Reduction* using Equation (2).

(2) $$logReduction=\log\left(N_{0}/N\right)$$

where $N_{0}$ and N  are the colony count (CFU/mL) before and after disinfection, respectively.

#### 4.3.2. Duty Cycle and Pulse Frequency

The duty cycle DC (%) is the proportion of turned-on time to a pulse cycle and the pulse frequency PF (Hz) is the number of pulse periods per second, as defined in Equations (3) and (4).

(3) $$DC=\frac{t_{on}}{t_{on}+t_{off}}\times 100\%$$

(4) $$PF=\frac{1}{t_{on}+t_{off}}$$

**Figure 11:**
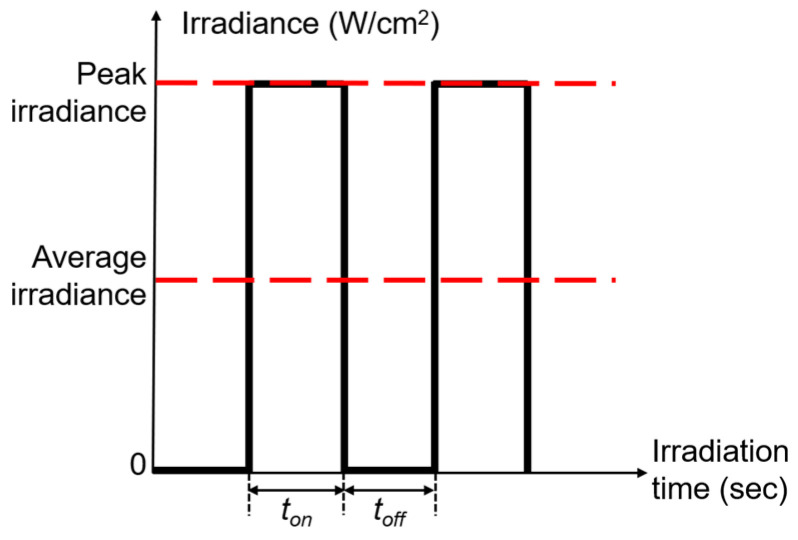
The illustration of the pulse signal.

As shown in Figure 11, $t_{on}$ and $t_{off}$ are the turned-on time and turned-off time of the LED during a pulse period. During the $t_{on}$, the output irradiance is the peak irradiance. During the $t_{off}$, the output irradiance is 0.

#### 4.3.3. Irradiation Dose, Irradiance, and Time

The conversion relations of the irradiation dose $D_{dose}$ (mJ/cm^2^), the peak irradiance $I_{peak}$ (mW/cm^2^), the average irradiance $I_{avg}$ (mW/cm^2^), and the irradiation time T (s) are shown in Equations (5) and (6).

(5) $$D_{dose}=I_{avg}\times T$$

(6) $$I_{avg}=I_{peak}\times DC$$

#### 4.3.4. Energy Efficiency

Energy efficiency is characterized by the log *Reduction* per unit of energy (LIE) (log/J), as defined in Equations (7) and (8) [44].

(7) LIE=R/E

(8) $$E=D_{dose}\times A$$

where R is the log *Reduction*, E is energy and in units of J, and A is the irradiation area (0.32 cm^2^ in this study).

#### 4.3.5. Dose Conservation Ratio

The amount of energy saved by the two irradiation methods that are being compared is described by the dose conservation ratio, which is specified in Equation (9).

(9) $$Dose\;conservation\;ratio=\frac{\left|D_{dose1}-D_{dose2}\right|}{\max\left\{D_{dose1},D_{dose2}\right\}}$$

where $D_{dose1}$ and $D_{dose2}$ represent the irradiation dose of the two methods compared.

#### 4.3.6. Model for Bacterial Inactivation Kinetics

Kumar et al. [41] found that the Hom model [16] can best fit the inactivation rate with the irradiation dose and be transformed into the most widely used first-order kinetics and the Weibull model [57]. The Hom model is in the form of Equation (10).

(10) $$R=a{D_{dose}}^{b}$$

Substituting Equation (5) into Equation (10) can obtain

(11) $$R=a{\left(I_{avg}\times T\right)}^{b}$$

According to the prior knowledge, one can assume that T and I have different exponent weights b on the reduction. The coefficient a signifies the proportional relationship between the left and right sides of the equation; hence, distinct weights are not introduced. The 2-D Hom model can be proposed and defined as follows:

(12) $$R=a({I_{avg}}^{b_{1}}\times T^{b_{2}})$$

For the desired reduction $R_{d}$, Equation (12) can be rewritten as

(13) $$T=\log_{b2}\frac{\left(R_{d}/a\right)}{{I_{avg}}^{b1}}$$

Combining Equation (5) and Equation (13) can obtain

(14) $$D_{dose}=I_{avg}\times \log_{b2}\frac{\left(R_{d}/a\right)}{{I_{avg}}^{b1}}$$

By minimizing Equation (14), the optimal combination of the average irradiance and irradiation time can be obtained. The 2-D model can balance the irradiance and time to improve energy efficiency and save time costs as much as possible.

### 4.4. ROS Assay

Intracellular ROS levels were measured with the ROS fluorescent probe (DCFH-DA assay kit, Beyotime Institute of Biotechnology, China). This probe can convert to highly fluorescent 2′,7′-dichlorofluorescein (DCF) by ROS. The bacterial cells were separated from the growth medium through centrifugation and were subsequently resuspended in a 500-fold dilution of the probe (in a final concentration of 10 μM/L) in PBS. After incubating at 37 °C in a shaker for 20 min, the suspension was centrifuged three times to remove the redundant probe. Then, the suspension was seeded into a 96-well plate and irradiated by aBL. The control group was kept in the dark. Immediately after the treatment, the intracellular ROS levels were measured with a spectrophotometer (Infinite M1000 PRO, Tecan, Switzerland) at an excitation wavelength (Ex) of 488 nm and emission wavelength (Em) of 525 nm.

### 4.5. Bacterial Enumeration

After aBL irradiation, 0.1 mL of the treated suspension was serially diluted 10-fold in 0.9 mL PBS, and the appropriate diluents were spread-plated onto LBA plates. The plates were incubated at 37 °C for 24 h. Typical white colonies were enumerated.

### 4.6. Statistical Analysis

All the experiments were performed in triplicate and each experiment was repeated thrice. The data are expressed as means ± the standard deviations and were statistically analyzed using a two-tailed *t*-test performed with a 95% confidence interval using Microsoft Excel (Version 15.30; Microsoft Corporation, Redmond, WA, USA). The differences between the groups were considered significant at *p* < 0.05. Surface and curve fitting was performed with the help of MATLAB R2020a (The MathWorks, Inc., Natick, MA, USA).

## 5. Conclusions

The effects of irradiance and irradiation time on the energy efficiency during aBL inactivation were explored to develop a more efficient and energy-saving aBL irradiation method. It was found that *E. coli* is more sensitive to aBL with a low duty cycle and a lower irradiation dose is required to achieve the same reduction. Compared with continuous exposure, $DC_{12.5\%}$ pulsed exposure can save ~54% of the energy and achieve 4.86 times higher energy efficiency. The improvement can be attributed to the enhancement of ROS generation. The group with a $DC_{25\%}$ and $T_{60min}$ generated the highest ROS concentration, which was 1.95 times higher than the group with a $DC_{100\%}$ and $T_{15min}$. The proposed 2-D Hom model (the exponent of *I* is 1.083; the exponent of *T* is 1.677) fitted the irradiance-time-reduction data. The model can obtain the optimal combination of the average irradiance and time according to the desired reduction and indicates that the irradiation time will cause a larger change in the reduction. These results are consistent with previous studies in both the field of UV inactivation and aBL inactivation. In terms of pulse frequency, when the duty cycle is 25%, a higher pulse frequency can achieve a better inactivation effect. Thus, for energy conservation purposes, it is better to use pulse exposure with a lower average irradiance for a longer period of time. However, there is a certain minimum irradiance and dose threshold for effective inactivation. The method of application of PWM signals to generate the desired irradiance can improve the inactivation efficiency and is easy to integrate into existing LED lighting systems, which helps promote the application of the aBL technique and saves energy during long-term lighting operation.

## Figures and Tables

**Table 1:** Fitting parameters and goodness of Hom model and calculated dose for 3 and 5 log reductions.

DC	Irradiance (mW/cm^2^)	a	b	R^2^	RMSE	T_3d_ (min)	T_5d_ (min)	D_3d_ (J/cm^2^)	D_5d_ (J/cm^2^)
12.5%	119.88	4.47 × 10^−6^	2.20	0.9800	0.1920	61.88	78.05	445.06	561.38
25.0%	239.76	5.09 × 10^−7^	2.43	0.9885	0.2746	42.05	51.87	604.88	746.12
50.0%	479.51	4.77 × 10^−6^	2.00	0.9943	0.1946	27.56	35.58	792.88	1023.61
75.0%	719.27	2.64 × 10^−5^	1.72	0.9920	0.2826	20.07	27.01	866.25	1165.60
100.0%	959.02	1.40 × 10^−6^	2.12	0.9949	0.2605	16.65	21.19	958.33	1219.02

## Data Availability

The data presented in this study are available on request from the corresponding author.
